# Enhanced electrochemical performance of lithia/Li_2_RuO_3_ cathode by adding tris(trimethylsilyl)borate as electrolyte additive

**DOI:** 10.1038/s41598-020-70333-2

**Published:** 2020-08-11

**Authors:** Byeong Gwan Lee, Yong Joon Park

**Affiliations:** Department of Advanced Materials Engineering, Kyonggi University, 154-42, Gwanggyosan-ro, Yeongtong-gu, Suwon-si, 16227 Gyeonggi-do Republic of Korea

**Keywords:** Batteries, Batteries

## Abstract

In this study, we used tris(trimethylsilyl)borate (TMSB) as an electrolyte additive and analysed its effect on the electrochemical performance of lithia-based (Lithia/Li_2_RuO_3_) cathodes. Our investigation revealed that the addition of TMSB modified the interfacial reactions between a lithia-based cathode and an electrolyte composed of the carbonate solvents and the LiPF_6_ salt. The decomposition of the LiPF_6_ salt and the formation of Li_2_CO_3_ and -CO_2_ was successfully reduced through the use of TMSB as an electrolyte additive. It is inferred that the protective layer derived from the TMSB suppressed the undesirable side reaction associated with the electrolyte and superoxides (O^-^ or O^0.5-^) formed in the cathode structure during the charging process. This led to the reduction of superoxide loss through side reactions, which contributed to the increased available capacity of the Lithia/Li_2_RuO_3_ cathode with the addition of TMSB. The suppression of undesirable side reactions also decreased the thickness of the interfacial layer, reducing the impedance value of the cells and stabilising the cyclic performance of the lithia-based cathode. This confirmed that the addition of TMSB was an effective approach for the improvement of the electrochemical performance of cells containing lithia-based cathodes.

## Introduction

One of the most important issues relating to lithium-ion batteries (LIBs) is the development of new cathodes with higher energy densities than commercially used LiCoO_2_, Li(Ni, Co, Mn)O_2_ (NCM), and Li(Ni, Co, Al)O_2_ (NCA)^1–7^. Fundamentally, the capacity of most cathodes can be attributed to the cationic redox of transition metals such as Co, Ni, and Mn in the structure. Therefore, it is necessary to increase the amount of transition metals (per weight or volume) in the cathode structure to improve its energy density, which is not an easy task considering the crystal structure of transition metal oxides. However, if the redox reaction of the anions (such as oxygen ions) can contribute to the capacity of the cathode, this problem can be overcome. The anions are much lighter than general transition ions, therefore the cathodes can provide much higher capacity (per weight) and energy density compared to cases relying on only cationic redox reactions. Subsequently, cathodes based on anionic redox reactions have been attractive research topics of late. As promising new cathode materials, Li–Nb–Mn–O, Li–Mn–O, and Li–Ru–M–O (M = Sn, Nb) have been studied for years^8–13^. These materials have shown high capacity—up to approximately 300 mA·g^−1^ based on anionic redox associated with oxygen as well as cationic redox related to transition metals. However, their sluggish kinetics and structural instability have resulted in an inferior rate capacity and cyclic performance compared to commercial cathodes^8–13^.

Lithia (Li_2_O)-based materials are also one of the new candidates for high-capacity cathodes based on anionic redox reactions^14–20^. In fact, most of the new cathodes such as Li–Nb–Mn–O, Li–Mn–O, and Li–Ru–M–O (M = Sn, Nb) provide capacity primarily based on the cationic redox reaction, although some portion of the capacity is clearly attributed to the anionic redox reaction. By contrast, the capacity of lithia-based cathodes is mostly related to the anionic redox reaction between oxygen ions (O^2-^) in the structure and superoxides (O^x-^, 0.5 ≤  X  < 2). Moreover, their cyclic performance is stable if the capacity is limited to avoid the formation of gaseous oxygen or the generation of superoxides above a threshold. However, lithia-based cathodes require suitable catalysts (sometimes called dopants) because lithia is not in itself electrochemically active. Therefore, the research of lithia-based cathodes to date has focused on the exploration of good catalysts for lithia and the combination between the catalysts and lithia ^14–20^. However, a fact that cannot be overlooked is that lithia-based cathodes are vulnerable to organic electrolytes, as are most cathodes. In particular, the basic reaction mechanism of lithia-based cathodes that form superoxides (O^x-^, 0.5 ≤  X  < 2) in the structure during cycling may promote undesirable reactions between liquid electrolytes. Therefore, research to stabilise the interface between the cathode and the electrolyte is essential in order to obtain stable electrochemical performance from lithia-based cathodes. Nevertheless, such studies are still at a rudimentary stage.

A representative approach to improve interfacial stability between a cathode and an electrolyte is surface coating of the cathode, which is widely used for commercial LIBs^21–27^. However, it is difficult to apply to lithia-based cathodes because a coating process utilising heat-treatment may significantly deteriorate lithia-based cathodes due to the extremely reactive lithia. Whereas, the use of electrolyte additives may be a promising solution to the interfacial problem of lithia-based cathodes^28–29^. The additives dissolve in the decomposed electrolyte during cycling to form a protective layer on the surface of the cathode. Since they are not used directly on the cathode, they can be applied to highly reactive lithia-based cathodes without any trouble.

In this study, tris(trimethylsilyl)borate (TMSB) was introduced as an electrolyte additive for a lithia-based cathode. TMSB reportedly forms a stable film layer on the surface of the cathode, which stabilises the cathode/electrolyte interface^30–34^. For a lithia-based cathode, lithia/Li_2_RuO_3_ nanocomposites were used due to their high capacity and good cycle life^20^. The most anticipated effect of this study was the increase in the available capacity of the lithia/Li_2_RuO_3_ nanocomposites. In general, the capacity of commercial cathodes was not critically affected by the electrolyte additive, but the effect of the additive significantly enhanced the rate capability, cyclic performance, and efficiency of the cells. By contrast, some lithia-based cathodes showed improved available capacity through the addition of electrolyte additives^28–29^, a fairly unique outcome. In this work, the electrochemical performance of lithia/Li_2_RuO_3_ nanocomposites through the addition of TMSB was carefully characterised.

### Electrochemical performance

**Figure 1:**
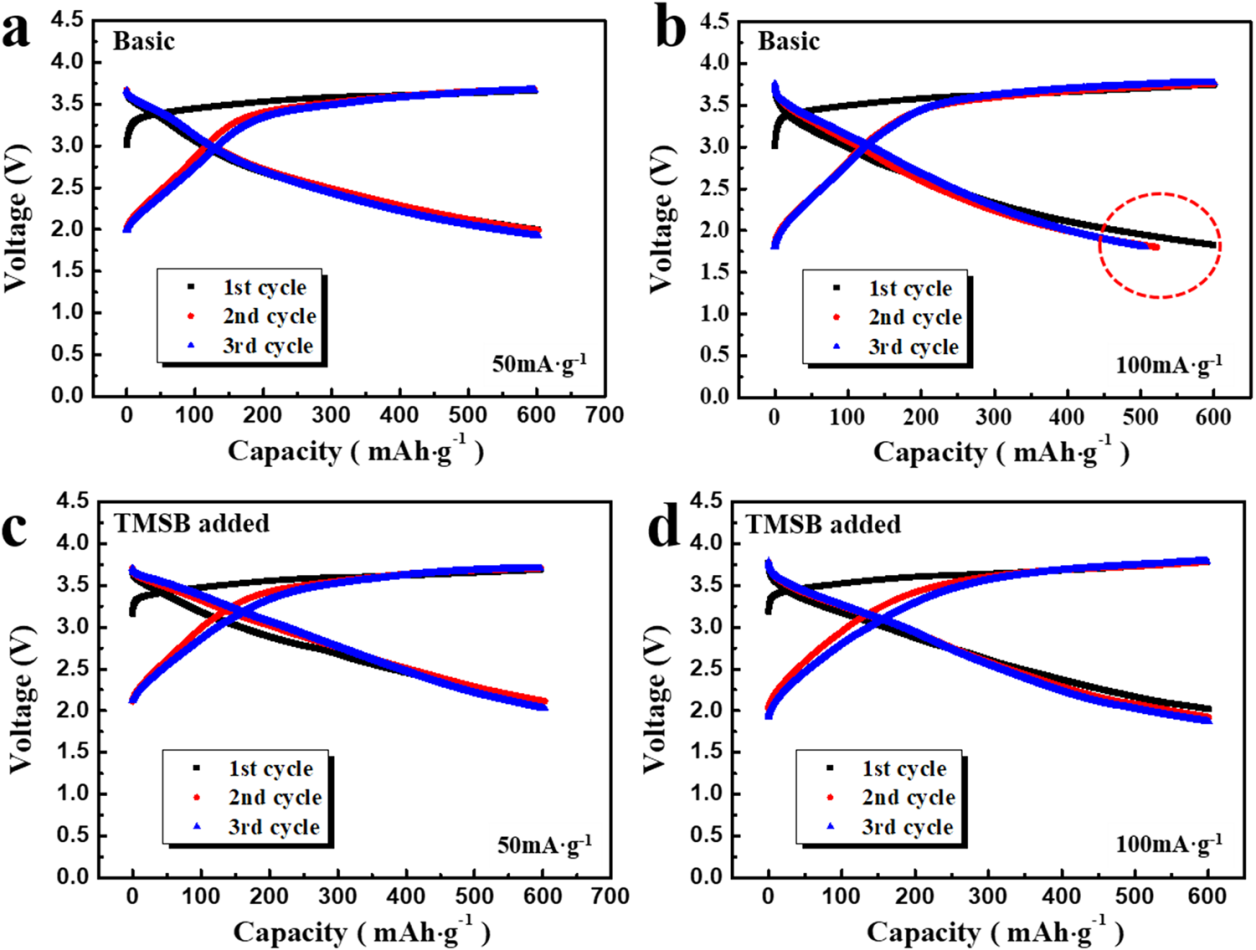
Charge–discharge profiles of the cells containing lithia/Li_2_RuO_3_ nanocomposite with limited capacity of 600 mAh·g^−1^, measured **(a)** at 50 mA·g^−1^ using the basic electrolyte, **(b)** at 100 mA·g^−1^ using the basic electrolyte, **(c)** at 50 mA·g^−1^ using TMSB added electrolyte, and **(d)** at 100 mA·g^−1^ using TMSB added electrolyte.

For convenience, the electrolyte without TMSB was named ‘basic’ electrolyte in this work. To evaluate the influence of TMSB on the electrochemical properties, the discharge capacity, and the cyclic performance of the cells containing lithia/Li_2_RuO_3_ nanocomposites, the cathodes were tested using the basic and TMSB added electrolytes. Figure 1 shows the charge–discharge profiles of the cells for three cycles. To compare the available capacity of the cells due to the difference in electrolytes, the measuring capacity was limited to 600 mAh·g^−1^ based on the lithia weight of the cathodes. The capacity of lithia-based cathodes was limited during cycling to afford them an obtainable available capacity whilst avoiding the capacity fading attributable to cathode instability due to overcharging^15–20^. At a current density of 50 mA·g^−1^, the cell using the basic electrolyte showed a stable charge–discharge profile and retained its limited capacity of 600 mAh·g^−1^ for three cycles (Fig. 1a). The initial charge profile was different from the subsequent charge profiles, inferring that an irreversible reaction of the cathode had occurred during the initial cycle. When the current density increased to 100 mA·g^−1^, the cell using the basic electrolyte did not retain its limited capacity (600 mAh·g^−1^) during cycling. As shown in Fig. 1b, the capacity decreased during the second cycle, indicating that the available capacity of the cell using the basic electrolyte was below 600 mAh·g^−1^. By contrast, as shown in Fig. 1c, d, the cells using TMSB added electrolyte showed a stable charge–discharge profile over three cycles at 50 mA·g^−1^ as well as at 100 mA·g^−1^. Although the overpotential of the cells increased at a current density of 100 mA·g^−1^ compared to those at 50 mA·g^−1^, the limited capacity of the cells (600 mAh·g^−1^) was maintained.

Figure S1 shows the comparison of the cyclic performance of the cells using the basic and TMSB added electrolytes for 50 cycles with a limited capacity of 600 mAh·g^−1^. The cells using the basic electrolyte maintained their capacity for only 12 cycles and 1 cycle at current densities of 50 and 100 mA·g^−1^, respectively (Fig. S1a, b). However, when the TMSB was added to the electrolyte, the cells maintained their limited capacity for the full 50 cycles, indicating that the available capacity of the lithia/Li_2_RuO_3_ nanocomposites was increased by the use of the TMSB additives (Fig. S1c, d).

Fundamentally, the capacity of a lithia-based cathode is attributed to the redox reaction of lithia (Li_2_O). The oxygen ions (O^2-^) in the lithia are oxidised to superoxides (O^-^ or O^0.5-^) during charging, maintaining a condensed phase in the oxide structure^17–20^, and the superoxides are reduced during discharging. When the lithia-based cathodes are overcharged, the oxygen ions in the cathode can be oxidised to gaseous oxygen, resulting in the evaporation of oxygen and rapid capacity fading of the cathodes. Even if the oxygen ions are not oxidised to oxygen gas, the increase in superoxides (O^-^ or O^0.5-^) in the cathodes structure are also an unstable factor since reactive superoxides react easily with the electrolyte which leads to capacity fading of the cathodes. Therefore, the capacity of a lithia-based cathode should be limited during cycling to avoid side reactions derived from superoxides as well as oxygen generation. The catalysts in a lithia-based cathode (such as Ru or Ru oxides in the lithia/Li_2_RuO_3_ nanocomposites) act as superoxide stabilisers. However, if the quantity of superoxide in the structure increases due to overcharging, the catalyst may reach its limit, and undesirable side reactions by superoxides can no longer be prevented.

As shown in Fig S1a, b, the unstable capacity of the cells using the basic electrolyte means that oxygen ions in the lithia/Li_2_RuO_3_ nanocomposite structures are overly oxidised to superoxides (O^-^ or O^0.5-^). Thus, when the cathode is charged to 600 mAh·g^−1^, the quantity of them is beyond the limit at which the superoxides can retain their phase in the structure without undesirable reactions. By contrast, the stable cyclic performance of the cells using TMSB added electrolyte indicates that the superoxides in the cathode charged to 600 mAh·g^−1^ are stabilised by the effect derived from the TMSB (Fig S1c, d). It should be considered that the quantity of superoxides in the cathodes was not changed by the use of TMSB, as the redox reaction of the oxygen ions occurs to provide the same 600 mAh·g^−1^ capacity. Therefore, it is reasonable to suggest that the superoxides formed by the charging process were stabilised in the structure due to the role of TMSB.

**Figure 2:**
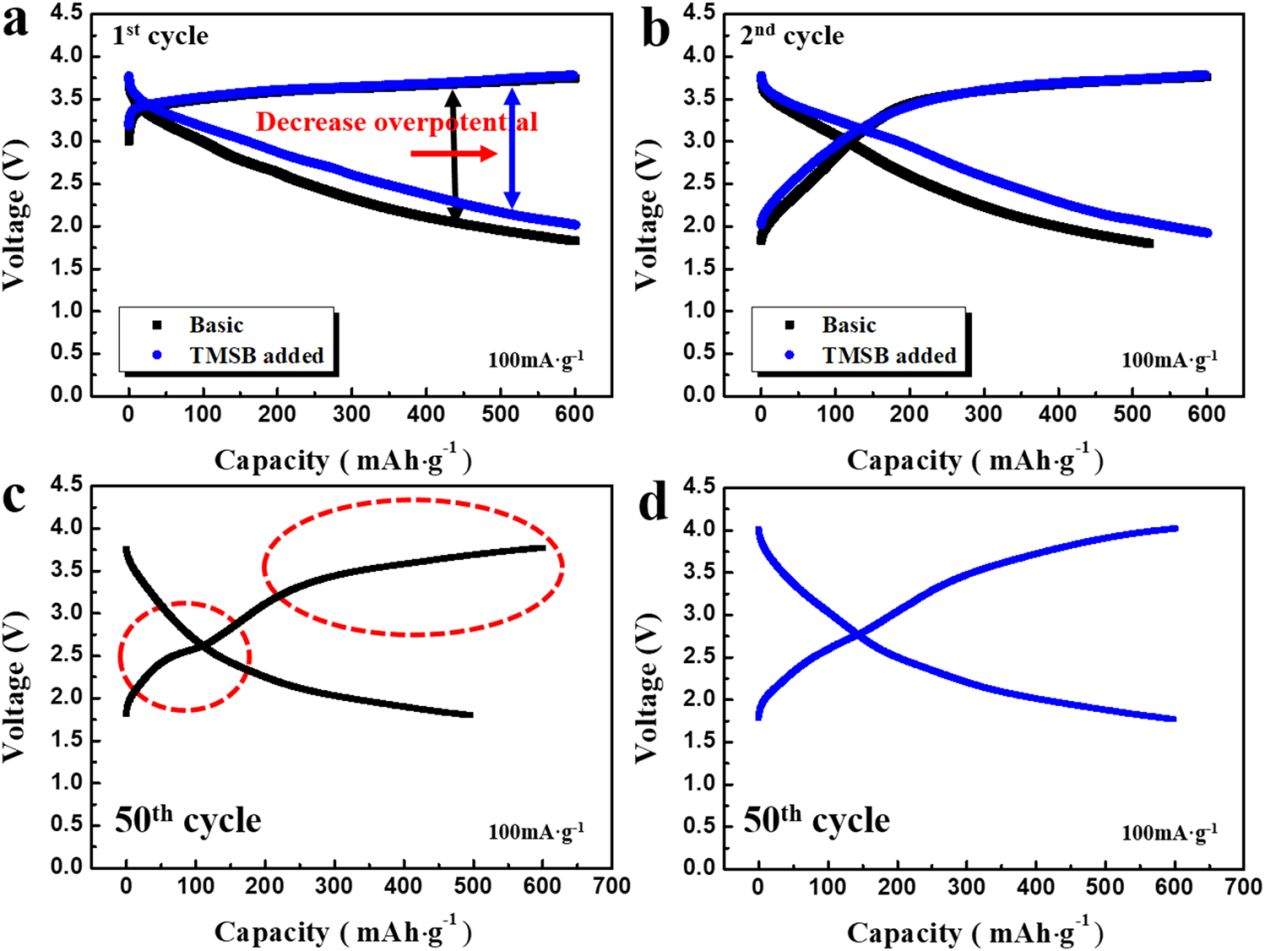
Voltage profiles of cells containing lithia/Li_2_RuO_3_ nanocomposite at a current density of 100 mA·g^−1^ with a limited capacity of 600 mAh·g^−1^**(a)** the first cycle using the basic and TMSB added electrolytes, **(b)** the second cycle using the basic and TMSB added electrolytes, **(c)** the 50th cycle using the basic electrolyte, and **(d)** the 50th cycle using TMSB added electrolyte.

Figure 2a, b compare the first and second charge–discharge profiles of the cells using the basic and TMSB added electrolytes. Regardless of the type of electrolyte used, the shape of the profiles was similar. However, the overpotential of the cells was reduced by the addition of TMSB, suggesting that the TMSB added electrolyte may reduce resistance factors during cycling. It is notable that the shape of the charge–discharge profiles after 50 cycles changed significantly due to the use of the TMSB additive. As shown in Fig. 2c, when the basic electrolyte was used, the charge–discharge profile at the 50th cycle exhibited two distinct steps, which may be associated with the degradation of lithia-based cathodes during cycling. By contrast, when the TMSB added electrolyte was used, the charge–discharge curve after 50 cycles showed a relatively smooth profile without any clear voltage step, as shown in Fig. 2d. Although the overpotential increased, it appears that the cathodes were not critically damaged during cycling.

### Characterization of interfacial reaction

From the results of the charge–discharge profile and cyclic performance, it was confirmed that the addition of TMSB in the electrolyte enhanced the electrochemical performance of the cells containing lithia/Li_2_RuO_3_ nanocomposites. It was expected that the TMSB dissolved in the electrolyte would decompose (oxidise) during the charging process to form a stable protective layer on the surface of the cathodes. To observe the morphological change caused by the addition of TMSB, lithia-based cathodes were analysed using scanning electron microscopy (SEM) and transmission electron microscopy (TEM). Figure S2 shows the SEM images of the cathodes composed of lithia/Li_2_RuO_3_ nanocomposites and carbon nanotubes (CNTs) before and after cycling. As shown in Fig. S2a, the particles of the lithia/Li_2_RuO_3_ nanocomposites and CNTs seemed to have agglomerated each other before the electrochemical test. Interestingly, the agglomeration of the particles was considerably suppressed after the first cycle, as shown in Fig. S2b, c. This may have been associated with the volume contraction/expansion of the lithia (Li_2_O) due to lithiation/delithiation during cycling. After 50 cycles, the cathodes appeared to be composed of small-sized nanoparticles, as shown in Fig. S2d, e.

**Figure 3:**
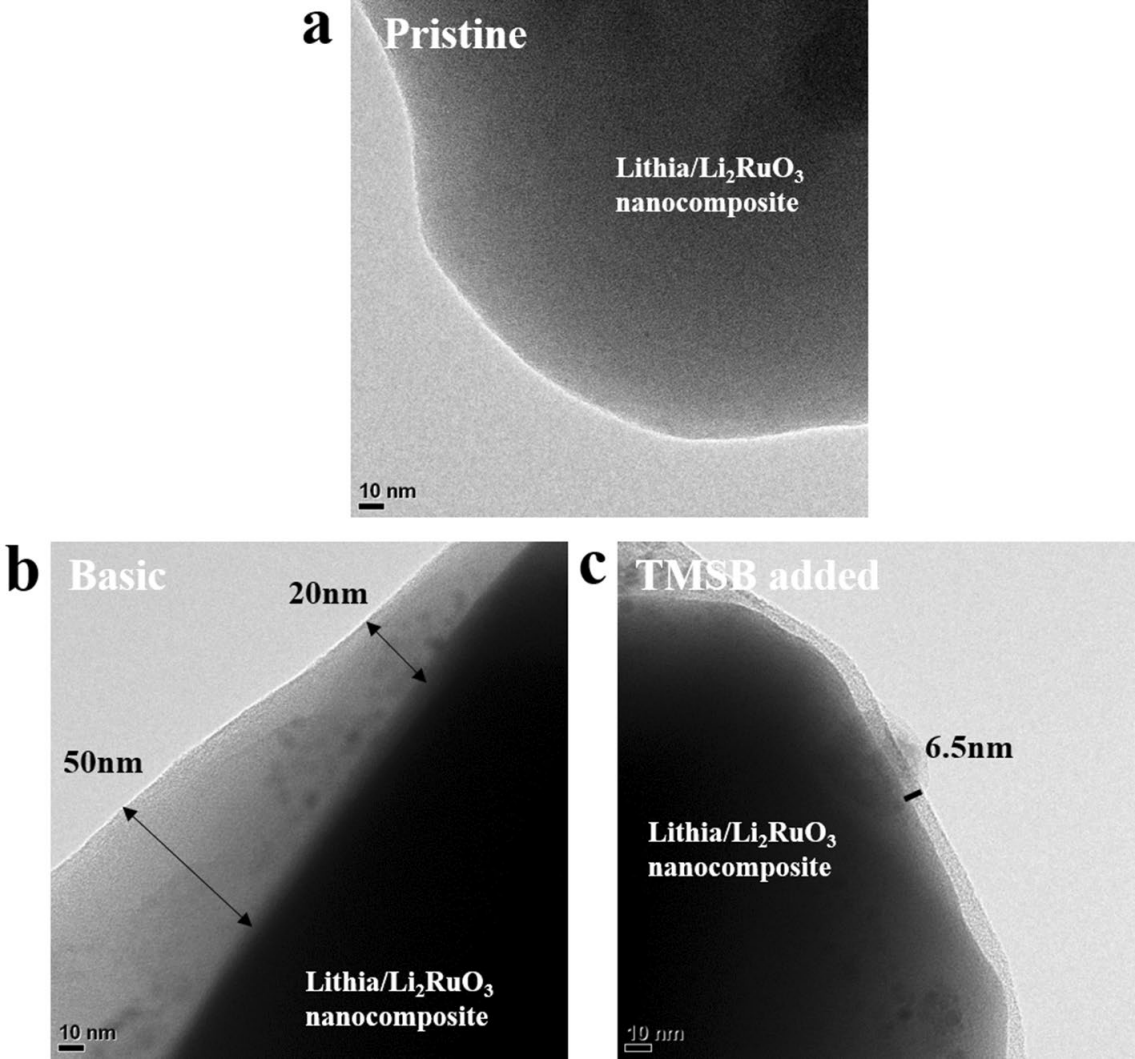
TEM images of the electrodes containing lithia/Li_2_RuO_3_ nanocomposite **(a)** before the electrochemical test, **(b)** after 50 cycles using the basic electrolyte, and **(c)** after 50 cycles using TMSB added electrolyte. (The cycle test was performed using a limited capacity of 600 mAh·g^−1^).

To analysis the surface of the cathode in detail, the TEM images of the pristine (before the electrochemical test) and cycled cathode-particles using the basic and TMSB added electrolytes were observed. As shown in Fig. 3a, the surface of the cathode-particle before the electrochemical test was clear without a heterogeneous surface layer. However, the surface was dramatically changed after 50 cycles with the limited capacity of 600 mAh·g^−1^ (current density = 100 mA·g^−1^). As shown in Fig. 3b, the cathode-particle cycled with the basic electrolyte was covered by a thick interfacial layer (20–50 nm). This kind of interfacial layer can be formed from the decomposition of a solvent such as ethylene carbonate (EC) and a salt such as LiPF_6_ in the electrolyte due to their reaction with the cathode surface. In fact, it has been known that the decomposition of an electrolyte occurs mainly at high voltages (over the range 4.2–4.3 V)^30–34^. Given the fact that most of the charging process of the cells containing lithia/Li_2_RuO_3_ nanocomposites takes place below 4.0 V, it is doubtful that this thick interfacial layer can be attributed to the decomposition of the electrolyte. However, the lithia-based cathode generates many superoxides with high reactivity during the charging process. The superoxides (O^-^ or O^0.5-^) can activate the decomposition of the electrolyte while consuming themselves by reaction with the solvent or salt. As shown in Figs. 1 and S1, the cells using the basic electrolyte could not retain their limited capacity of 600 mAh·g^−1^ during cycling. As mentioned, this shows that cells charged to 600 mAh·g^−1^ were in an overcharged state and the quantity of superoxides in the cathodes exceeded the critical point at which they could stably exist in the structure of the cathode without side reactions. Therefore, the thick interfacial layer in the cycled cathode (Fig. 3b) was due primarily to the undesirable reactions between the electrolyte and superoxides in the cathode structure.

Notably, the interfacial layer was dramatically reduced by the addition of TMSB in the electrolyte. As shown in Fig. 3c, the cathode cycled 50 times using TMSB added electrolyte exhibited a relatively thin interfacial layer. We inferred that the TMSB in the electrolyte formed a stable protection layer, which successfully prevented the progress of undesirable interfacial reactions related to superoxides. Therefore, the loss of superoxides in the structure can be prevented during cycling, even if their quantity reaches the point at which they cannot retain stability using the basic electrolyte. The addition of TMSB to the electrolyte led to an increase in available capacity of the cells containing lithia-based cathodes (lithia/Li_2_RuO_3_ nanocomposites).

**Figure 4:**
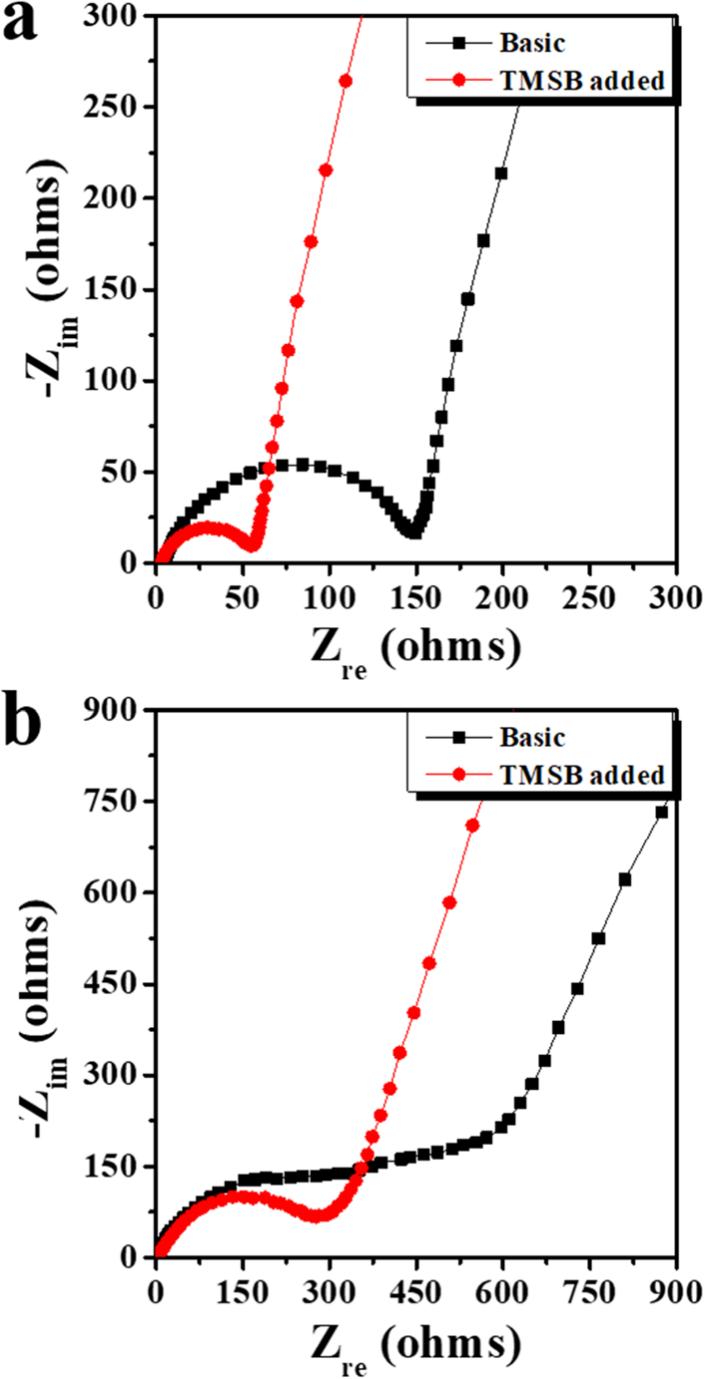
Nyquist plots of the cells containing lithia/Li_2_RuO_3_ nanocomposite cycled with a limited capacity of 600 mAh·g^−1^**(a)** after 1 cycle, and **(b)** after 50 cycles.

Figure 4 shows the Nyquist plots of the cells containing lithia/Li_2_RuO_3_ nanocomposites prepared using the basic and TMSB added electrolytes. The test was performed after the 1st and 50th cycles with the limited capacity of 600 mAh·g^−1^. As shown in Fig. 4, the semicircle size of the Nyquist plots of the cells was decreased by the use of TMSB regardless of the number of cycles, indicating that the impedance value of the cells was efficiently reduced by the effect of TMSB addition. The impedance value of the cells significantly increased after 50 cycles (Fig. 4b) compared to those after the first cycle (Fig. 4a). Nevertheless, the cell using TMSB added electrolyte showed much lower impedance value compared to the cell using the basic electrolyte. This result could be connected with the above TEM images as shown in Fig. 3b,c. The thick interfacial layer of the cathode cycled using a basic electrolyte may interfere with the movement of lithium ions and electrons during cycling, which results in the increase in impedance value of the cells. In contrast, the relatively thin and homogeneous interfacial layer of the cathode cycled using a TMSB added electrolyte leads to the much smaller impedance value than that of the cathode using the basic electrolyte.

To elucidate the TMSB effect, it is necessary to characterise the reaction products formed on the cathode/electrolyte interface. The electrodes containing lithia/Li_2_RuO_3_ nanocomposites were collected from the cells before and after cycles with a limited capacity of 600 mAh·g^−1^, and their X-ray photoelectron spectroscopy (XPS) profiles were analysed. Figure S3 shows the Si 2p and B 1s spectra of the electrodes cycled with the basic and TMSB added electrolyte. As shown in Fig. S3a, b, the spectra for the electrode cycled with the basic electrolyte did not exhibit clear peaks. By contrast, the spectra for the electrode after 1 cycle with TMSB added electrolyte showed clear peaks related to Si–O and B–O. Considering Si–O and B–O bonds derived from TMSB^33–35^, this confirmed that TMSB was decomposed during the initial cycle, and an interfacial layer was successfully formed on the surface of the cathode. The Si–O and B–O peaks were observed in the XPS spectra of the electrode after 50 cycles, as shown in Figs. S3c and S3d. However, their intensity did not increase much, indicating that further formation of the interfacial layer derived from TMSB was limited.

**Figure 5:**
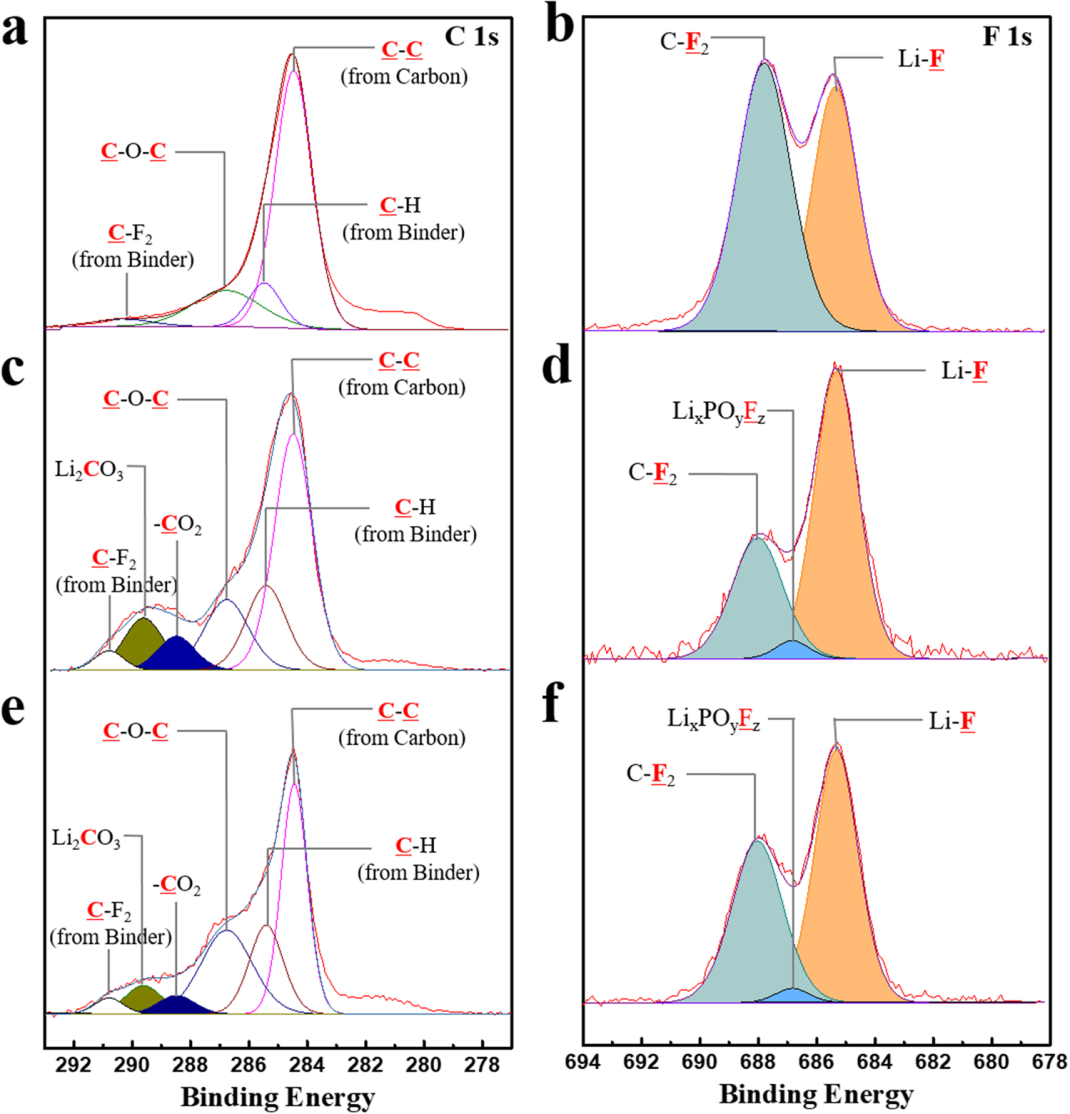
XPS profiles of the cathodes composed of lithia/Li_2_RuO_3_ nanocomposite before and after 1 cycle **(a)** C 1s spectrum of the electrode before the electrochemical test, **(b)** F 1s spectrum of the electrode before the electrochemical test, **(c)** C 1s spectrum of the electrode after 1 cycle using the basic electrolyte, **(d)** F 1s spectrum of the electrode after 1 cycle using the basic electrolyte, **(e)** C 1s spectrum of the electrode after 1 cycle using TMSB added electrolyte, and **(f)** F 1s spectrum of the electrode after 1 cycle using TMSB added electrolyte.

Figure 5 shows the C 1s and F 1s spectra of the electrode before and after one cycle. In the C 1s spectrum of the electrode before cycling (Fig. 5a), the C–C bond (~ 284.5 eV) corresponded with the conductive carbon in the electrode^36–37^, and C–H (~ 285.5 eV) and C–F_2_ (~ 290.8 eV) bonds could be attributed to the binder (PVDF). The C–O–C (~ 286.7 eV) bond was due to residual carbon and the binder^38^. In the F 1s spectrum of the electrode before cycling (Fig. 5b), the C–F_2_ (~ 688 eV) was associated with the PVDF binder in the electrode. A peak attributed to LiF (~ 685.1 eV) was also observed, which may have been related to the reaction with lithia (Li_2_O) in the electrode. After one cycle, the generation of new peaks were observed in the C 1s spectra of the electrodes, as shown in Fig. 5c,e. Li_2_CO_3_ (~ 289.6 eV) and –CO_2_ (~ 288.5 eV) were newly detected, formed from the undesirable reactions between the lithia-based cathode and the electrolyte (such as the decomposition of carbonate solvent in the electrolyte during cycling). The peak intensities of Li_2_CO_3_ and –CO_2_ were reduced by the addition of TMSB, as shown in Fig. 5e. This meant that the surface layer derived from TMSB could suppress undesirable interfacial reactions, which led to the low impedance value of the cells prepared using TMSB added electrolyte, as shown in Fig. 4a. The F 1s spectra of the electrode after one cycle also showed new peaks related to Li_x_PO_y_F_z_ (~ 686.9 eV), as shown in Fig. 5d,f, which were associated with the decomposition of the LiPF_6_ salt. The intensity ratio of the LiF/C–F_2_ peaks also increased after 1 cycle, which was attributed to the formation of LiF due to the reaction between the cathode (containing superoxides) and the LiPF_6_ salt or the PVDF binder. However, when TMSB added electrolyte was used, the intensity ratio of the LiF/C-F_2_ peaks was reduced ($\text{I}_{\text{LiF}}/\text{I}_{{\text{C-F}}_{2}}$ = 1.56) compared to that without the TMSB ($\text{I}_{\text{LiF}}/\text{I}_{{\text{C-F}}_{2}}$ = 2.35), suggesting that the formation of LiF was suppressed by the TMSB. This may be associated with the protection effect of the interfacial layer derived from the decomposition of TMSB.

**Figure 6:**
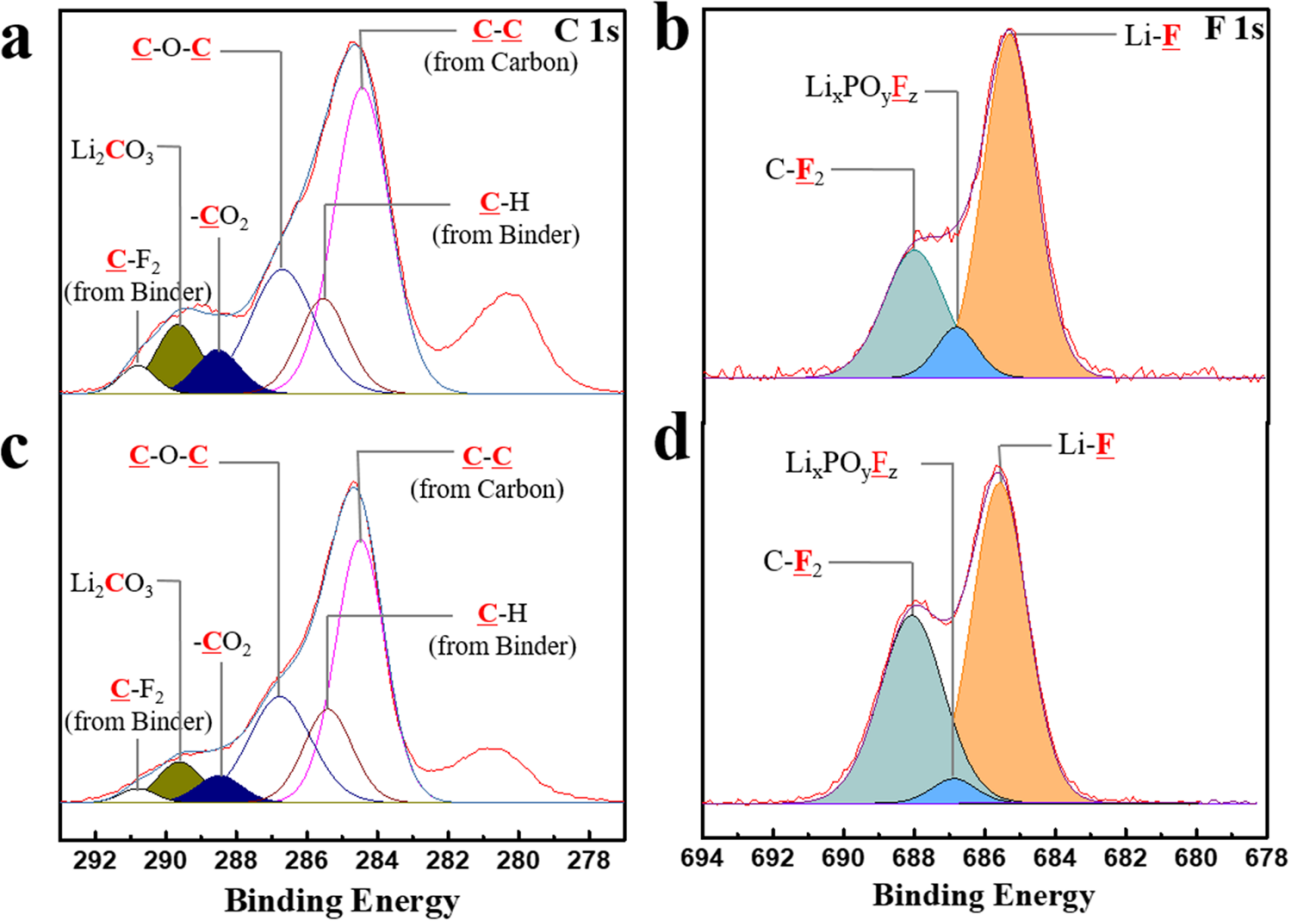
XPS profiles of the cathodes composed of lithia/Li_2_RuO_3_ nanocomposite after 50 cycles **(a)** C 1s spectrum of the electrode after 50 cycles using the basic electrolyte, **(b)** F 1s spectrum of the electrode after 50 cycles using the basic electrolyte, **(c)** C 1s spectrum of the electrode after 50 cycles using TMSB added electrolyte, and **(d)** F 1s spectrum of the electrode after 50 cycles using TMSB added electrolyte.

Figure 6 shows the C 1s and F 1s spectra of the electrode after 50 cycles. The C 1s spectra near the Li_2_CO_3_ and –CO_2_ peaks were enlarged, as shown in Fig. S4, to compare them after 1 and 50 cycles. Regardless of the type of electrolyte, the intensities of the peaks related to Li_2_CO_3_ and -CO_2_ seemed to increase a little over 50 cycles compared to those during the first cycle. However, it was not significant, as shown in Figs. 6a,c, and S4a–d. This meant that the decomposition of the carbonate solvent and side reactions for the formation of Li_2_CO_3_ did not proceed violently during subsequent cycling, after these reactions occurred during the first cycle. Instead, the intensity of the Li_x_PO_y_F_z_ peaks clearly increased (Fig. 6b,d) during the 50 cycles, indicating that a side reaction related to the LiPF_6_ salt actively progressed during cycling. Figure S5 compares the F 1s spectra near the Li_x_PO_y_F_z_ peak after 1 and 50 cycles. A substantial portion of the reaction products formed on the surface of the cathode during cycling could be attributed to the decomposition of the carbonate solvent [such as ethylene carbonate (EC)]. However, in our case, undesirable reactions related to the solvent were not activated during cycling after the formation of the interfacial layer containing Li_2_CO_3_ and –CO_2_ during the initial cycle. This may have been due to the low charging voltage (below 4.0 V) of the lithia-based cathodes.

**Figure 7:**
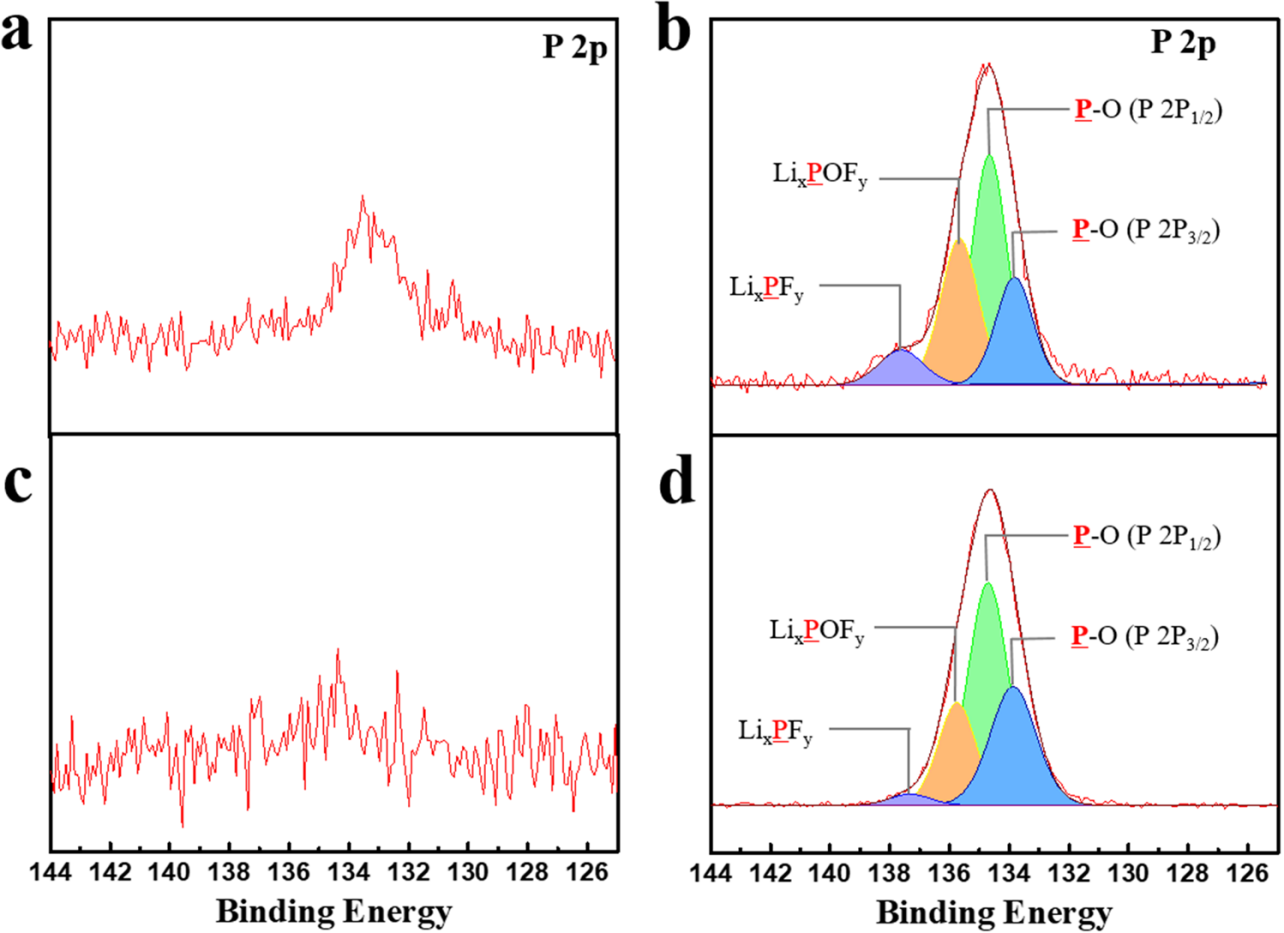
Comparison of the XPS profiles of the cathodes composed of lithia/Li_2_RuO_3_ nanocomposite after 1 and 50 cycles **(a)** P 2p spectrum of the electrode after 1 cycle using the basic electrolyte, **(b)** P 2p spectrum of the electrode after 50 cycles using the basic electrolyte, **(c)** P 2p spectrum of the electrode after 1 cycle using TMSB added electrolyte, and **(d)** P 2p spectrum of the electrode after 50 cycles using TMSB added electrolyte.

To further analyse the reaction related to the LiPF_6_ salt, the P 2p spectra of the electrode after 1 and 50 cycles were observed. As shown in Fig. 7a,c, the electrode after one cycle did not show clear peaks in the P 2p spectra. By contrast, as shown in Fig. 7b,d, the spectra of the electrode after 50 cycles exhibited large peaks comprising Li_x_POF_y_ (~ 135.5 eV), Li_x_PF_y_ (~ 138.8.9 eV), and phosphate (P–O) (~ 133.7 and 134.5 eV), which were generated from the decomposition of LiPF_6_^35^. This showed that the LiPF_6_ salt was continuously decomposed during cycling, which was due to superoxides in the charged lithia-based cathodes being more reactive with the LiPF_6_ salt than with the carbonate solvent during cycling.

It is instructive that the intensity of the Li_x_POF_y_ (~ 135.5 eV) and Li_x_PF_y_ (~ 137.8 eV) peaks were largely reduced by the addition of TMSB in the electrolyte (Fig. 7d), which indicated that addition of TMSB successfully suppressed the decomposition of the LiPF_6_ salt during cycling. Considering that the side reactions associated with the formation of Li_2_CO_3_ and –CO_2_ were also reduced by the use of TMSB, as shown in Fig. S4, it is clear that the undesirable reactions at the cathode/electrolyte interface were reduced due to the effect of TMSB. It was inferred that the protective layer derived from TMSB effectively reduced the reactivity of the superoxides in the lithia-based cathodes. The superoxides in the charged lithia-based cathode were consumed due to the side reactions with the electrolyte, which led to the capacity fading of the cathode during cycling. However, the addition of TMSB suppressed the loss of superoxides during cycling by reducing undesirable side reactions. As a result, the available capacity, which was obtained without capacity fading during cycling, was increased due to the effect of TMSB.

## Summary

**Figure 8:**
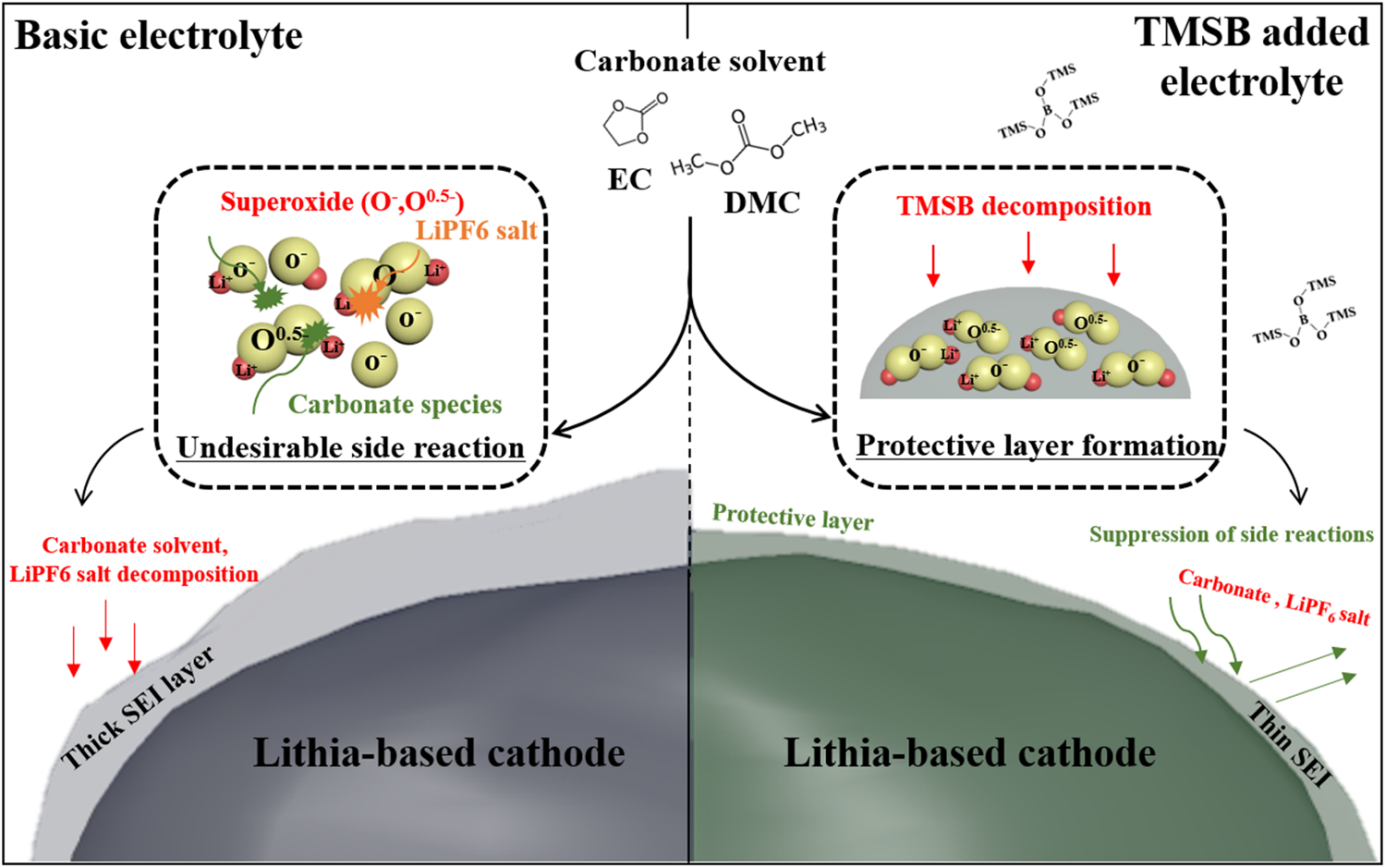
Schematic diagram illustrating the effect of TMSB added electrolyte on the electrochemical performance of the lithia-based cathode.

TMSB was introduced as an electrolyte additive to enhance the electrochemical performance of a lithia-based cathode composed of lithia/Li_2_RuO_3_ nanocomposites. Cells prepared using TMSB added electrolyte exhibited increased available capacity as well as improved cyclic performance compared to those using the basic electrolyte. An interfacial layer derived from the TMSB was formed during the initial cycle, which suppressed undesirable side reactions with the electrolyte (such as the decomposition of the LiPF_6_ salt and the formation of Li_2_CO_3_ and –CO_2_. We inferred that these side reactions were activated by the superoxides (O^-^ or O^0.5-^) formed during the charging process in the structure of the lithia-based cathode. Moreover, the suppression of side reactions also reduced the consumption of superoxides during cycling, which resulted in the increased available capacity of the lithia-based cathodes. Figure 8 summarised the effect of the TMSB additives on the electrochemical performance of lithia-based cathodes. This work explains the cause of the increased available capacity due to the use of electrolyte additives, and we trust that it will provide important insights for the active application of electrolyte additives in lithia-based cathodes in the future.

## Methods

To synthesise the Li_2_RuO_3_ used as a catalyst for lithia-based cathodes, pellets composed of a 1:1 (mol%) ratio of RuO_2_ (Alfa Aesar, 99.9%) and Li_2_CO_3_ (Aldrich, 99.99%) were sintered at 950 ℃ for 24 h. Next, a Li_2_RuO_3_ and lithia mixture comprising Ru content (f_Ru_ = Ru/(Ru + Li)) of 0.09 (mol%) was dispersed in butanol to obtain a uniform mixture (Lithia/Li_2_RuO_3_) which was then dried under vacuum at 90 ℃ for 24 h. It was then placed into a sealed zirconia milling container with zirconia balls [5 and 10 mm diameters (1:1 wt%)] in an Ar-glove box and ball-milled using a Planetary Mono Mill (Pulverisette 6, Fritsch) to obtain the lithia/ Li_2_RuO_3_ nanocomposites. The milling process was performed for 150 h (resting every 30 min after milling for one hour) at 600 rpm. The lithia/Li_2_RuO_3_ nanocomposite was formed and some of the Li_2_RuO_3_ was decomposed to Ru during this milling process^20^.

For the electrochemical test, the cathode material was mixed with 60 wt% active material (lithia/Li_2_RuO_3_ nanocomposite), 30 wt% Carbon nanotubes, and 10 wt% Polyvinylidene fluoride (PVDF) binder in *N*-methly-2-pyrrolidone (NMP), as the solvent, by ball-milling for 90 min. Next, the mixed slurry was cast on aluminium foil and dried under vacuum at 80 ℃ for 24 h. The cathode was assembled in a coin cell (2032-type) with Li metal as an anode, an electrolyte, and polypropylene (Celgard 2400) as the separator. For the ‘basic’ electrolyte, 1.0 M LiPF_6_ was dissolved in a mixture of EC and dimethyl carbonate (DMC) by volume ratio of 1:1. To obtain tris(trimethylsilyl)borate (TMSB, Aldrich, 99%) added electrolyte, 0.3 wt% of TMSB was dissolved into the electrolyte. The 2032-type coin cells were assembled in an Ar-glove box. The cycle test was conducted with current densities of 50 and 100 mA·g^-1^ and a cut-off voltage of 1.8–4.35 V using a WonATech voltammetry system. Impedance measurements were performed using an electrochemical workstation (Ametek, VersaSTAT 3) by applying an AC voltage with an amplitude of 5 mV over a frequency range of 0.1 Hz to 100 kHz. To observe the surface morphology before testing, after the first cycle, and after the 50th cycle, scanning electron microscopy (SEM, JEOL JSM-7610F PLUS) and transmission electron microscopy (TEM, JEOL JEM-2100F, Cs Corrector) were employed. Reaction products on the cathode surface were observed using XPS (Thermo Scientific K-Alpha^+^). XPS was conducted with monochromatised Al Kα radiation (hν = 1,486.6 eV). A vacuum transfer system was used to avoid moisture/air exposure of the sample during transfer to XPS. The obtained spectra were fitted using XPS peak software (Avantage Data System). The binding energy scale was calibrated using the C 1s peak at 284.6 eV from C–C peak.

## Supplementary information

Supplementary Information.
