# Supplementary material for: Enhanced electrochemical performance of lithia/Li2RuO3 cathode by adding tris(trimethylsilyl)borate as electrolyte additive
Source: Sci Rep. 2020 Aug 11;10:13498. doi: 10.1038/s41598-020-70333-2 (PMC7419494; doi:10.1038/s41598-020-70333-2)
Supplement: Supplementary file 1 — Supplementary Information. [file 41598_2020_70333_MOESM1_ESM.pdf]

Enhanced Electrochemical Performance of Lithia/Li_2RuO_3 Cathode by adding Tris(trimethylsilyl)borate as Electrolyte Additive

Byeong Gwan Lee and Yong Joon Park*

Department of Advanced Materials Engineering, Kyonggi University, 154-42, Gwanggyosan-Ro, Yeongtong-Gu, Suwon-Si, Gyeonggi-Do, 16227, Republic of Korea

* Corresponding author

Ph: +82-31-249-9769; E-mail: yjpark2006@kyonggi.ac.kr

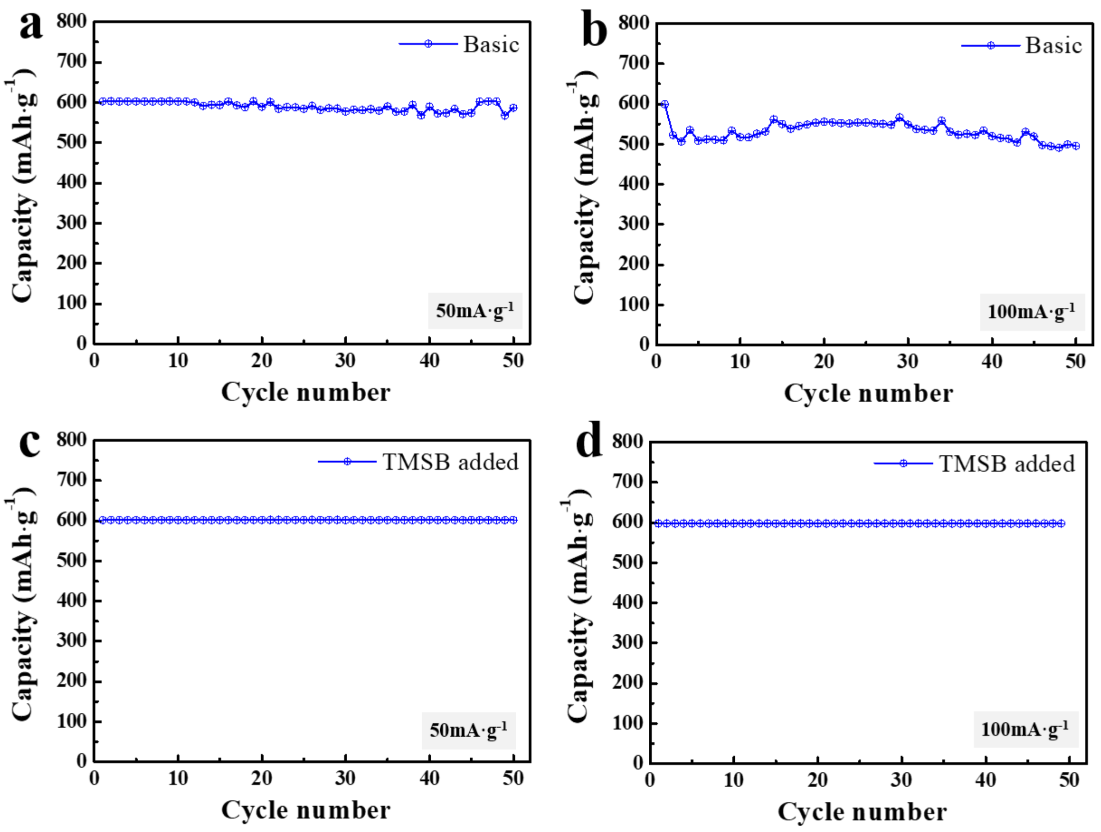

Figure S1. Cyclic performance of the cells containing a lithia/Li_2RuO_3 nanocomposite with a limited capacity of 600 mAh·g^{-1} measured (a) at 50 mA·g^{-1} using the basic electrolyte, (b) at 100 mA·g^{-1} using the basic electrolyte, (c) at 50 mA·g^{-1} using TMSB added electrolyte, and (d) at 100 mA·g^{-1} using TMSB added electrolyte.

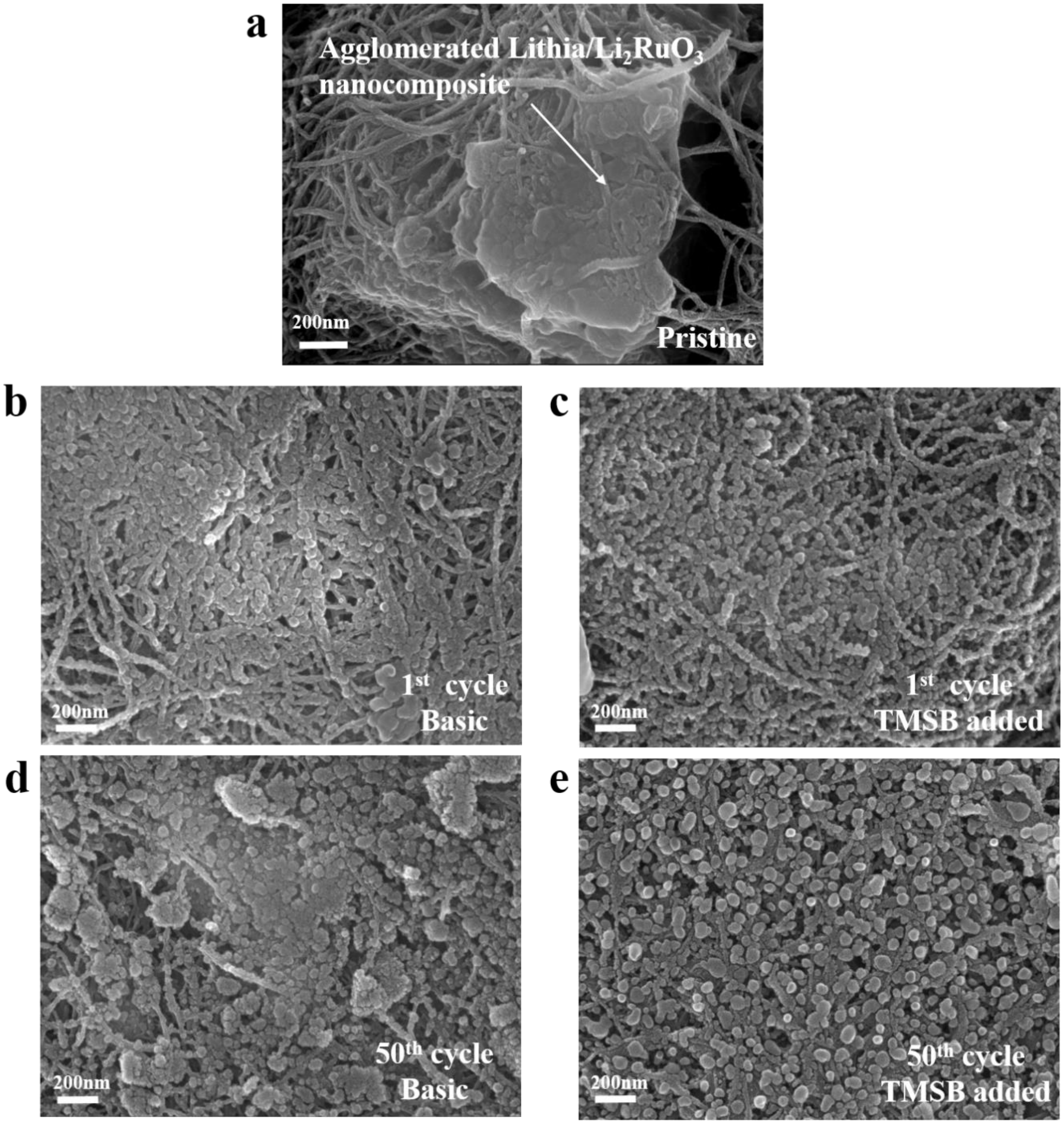

Figure S2. SEM images of the electrodes containing lithia/Li_2RuO_3 nanocomposite (a) before the electrochemical test, (b) after one cycle using the basic electrolyte, (c) after one cycle using TMSB added electrolyte, (d) after 50 cycles using the basic electrolyte, and (e) after 50 cycles using TMSB added electrolyte. (A cycle test was performed using a limited capacity of 600 $mAh \cdot g^{-1}$).

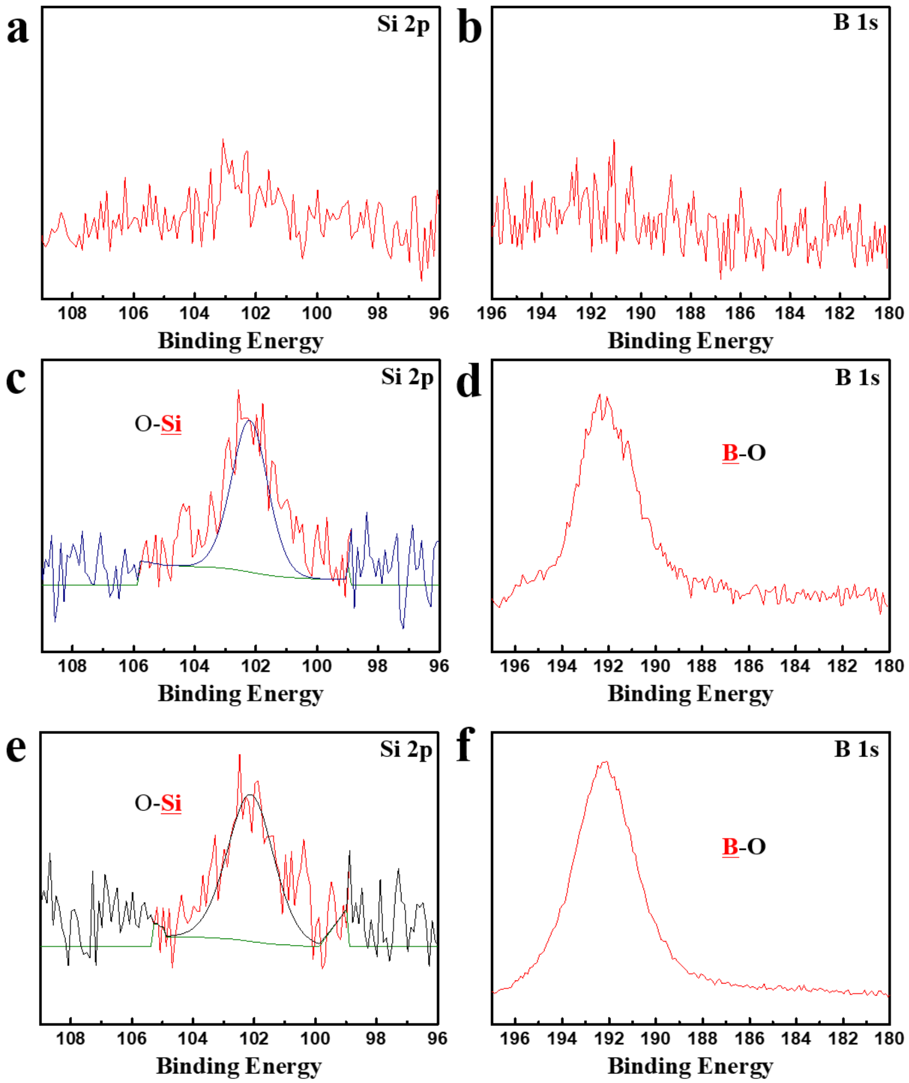

Figure S3. XPS profiles of the cathodes composed of lithia/Li_2RuO_3 nanocomposite after cycle (a) Si 2p spectrum of the electrode after 1 cycle using the basic electrolyte, (b) B 1s spectrum of the electrode after 1 cycle using the basic electrolyte, (c) Si 2p spectrum of the electrode after 1 cycle using TMSB added electrolyte, (d) B 1s spectrum of the electrode after 1 cycle using TMSB added electrolyte, (e) Si 2p spectrum of the electrode after 50 cycles using TMSB added electrolyte, and (f) B 1s spectrum of the electrode after 50 cycles using TMSB added electrolyte.

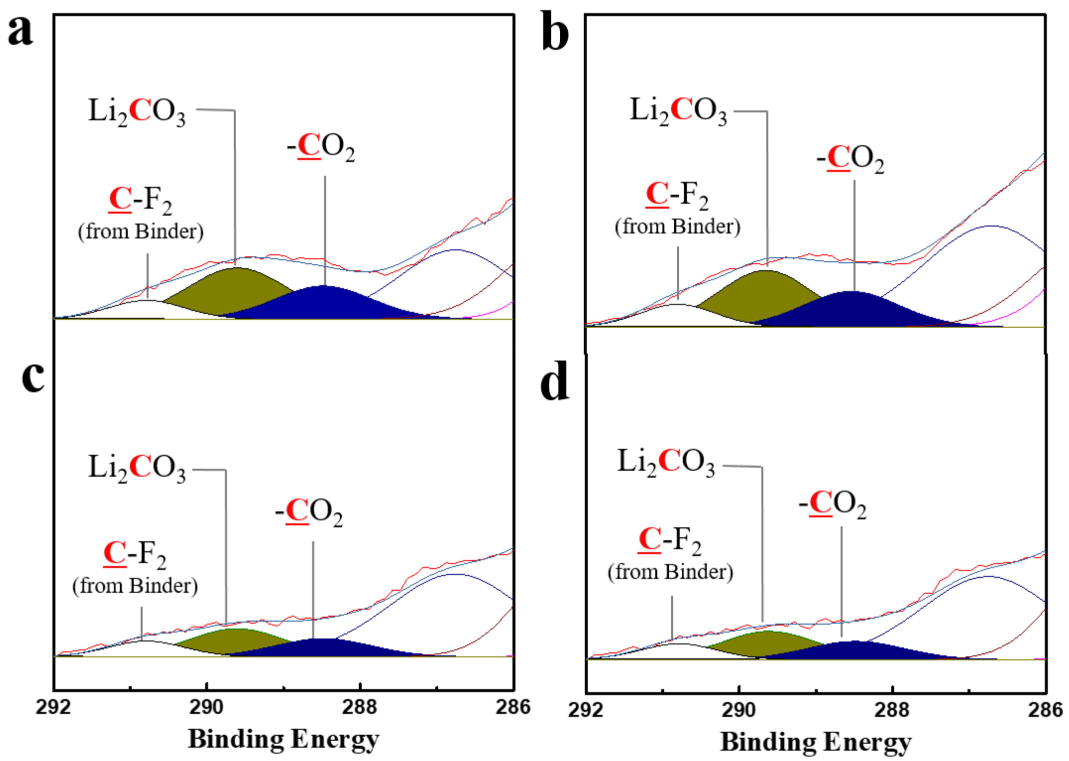

Figure S4. C 1s spectra of the cathodes composed of lithia/Li_2RuO_3 nanocomposite near Li_2CO_3 and $-CO_2$ peaks (a) after 1 cycle using the basic electrolyte, (b) after 50 cycles using the basic electrolyte, (c) after 1 cycle using TMSB added electrolyte, and (d) after 50 cycles using TMSB added electrolyte.

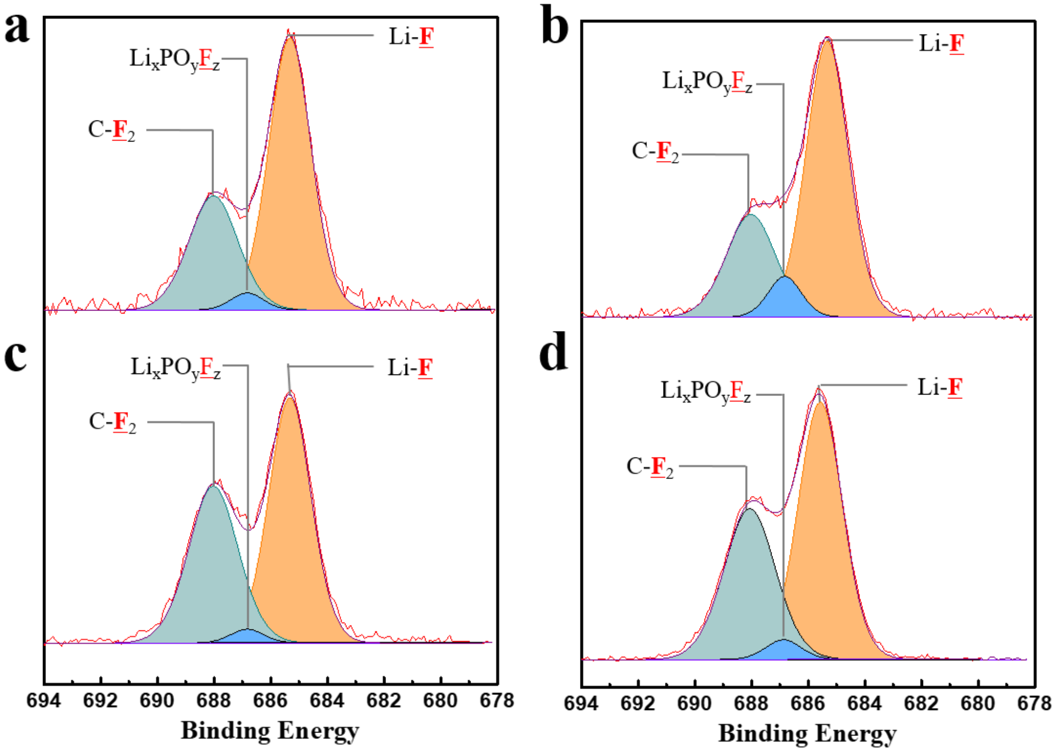

Figure S5. F 1s spectra of the cathodes composed of lithia/Li_2RuO_3 nanocomposite near $Li_xPO_yF_z$ peak (a) after 1 cycle using the basic electrolyte, (b) after 50 cycles using the basic electrolyte, (c) after 1 cycle using TMSB added electrolyte, and (d) after 50 cycles using TMSB added electrolyte.
